# Long-term survival outcomes of esophageal cancer after minimally invasive Ivor Lewis esophagectomy

**DOI:** 10.1186/s12957-022-02518-0

**Published:** 2022-02-25

**Authors:** Keouna Pather, Erin M. Mobley, Christina Guerrier, Rhemar Esma, Heather Kendall, Ziad T. Awad

**Affiliations:** 1 Department of Surgery, University of Florida College of Medicine – Jacksonville, Jacksonville, Florida, USA; 2 Division of General Surgery, Faculty Clinic, UF College of Medicine – Jacksonville, 653 West 8th Street, FC12, 3rd Floor, Jacksonville, FL 32209 USA; 3 UF Health Jacksonville, Jacksonville, Florida, USA

**Keywords:** Esophageal malignancy, Thoracic surgery, Thoracolaparoscopic esophagectomy, Ivor Lewis esophagectomy, Cancer survival

## Abstract

**Objectives:**

The aim of this study was to determine the long-term overall and disease-free survival and factors associated with overall survival in patients with esophageal cancer undergoing a totally minimally invasive Ivor Lewis esophagectomy (MILE) at a safety-net hospital.

**Methods:**

This was a single-center retrospective review of consecutive patients who underwent MILE from September 2013 to November 2017. Overall and disease-free survival were analyzed by Kaplan-Meier estimates, and hazard ratios (HR) were derived from multivariable Cox regression models.

**Results:**

Ninety-six patients underwent MILE during the study period. Overall survival at 1, 3, and 5 years was 83.2%, 61.9%, and 55.9%, respectively. Disease-free survival at 1, 3, and 5 years was 83.2%, 60.6%, and 47.5%, respectively. Overall survival (*p <* 0.001) and disease-free survival (*p* < 0.001) differed across pathological stages. By multivariable analysis, increasing age (HR, 1.06; *p* = 0.02), decreasing Karnofsky performance status score (HR, 0.94; *p* = 0.002), presence of stage IV disease (HR, 5.62; *p* = 0.002), locoregional recurrence (HR, 2.94; *p* = 0.03), and distant recurrence (HR, 4.78; *p* < 0.001) were negatively associated with overall survival. Overall survival significantly declined within 2 years and was independently associated with stage IV disease (HR, 3.29; *p* = 0.04) and distant recurrence (HR, 5.78; *p* < 0.001).

**Conclusion:**

MILE offers favorable long-term overall and disease-free survival outcomes. Age, Karnofsky performance status score, stage IV, and disease recurrence are shown to be prognostic factors of overall survival. Prospective studies comparing long-term outcomes after different MIE approaches are warranted to validate survival outcomes after MILE.

**Supplementary Information:**

The online version contains supplementary material available at 10.1186/s12957-022-02518-0.

## Highlights

- Minimally invasive Ivor Lewis esophagectomy offers favorable long-term outcomes.
- Overall survival was 62% at 3 years and 56% at 5 years at our safety-net hospital.
- Age, Karnofsky score, stage IV, and disease recurrence affect overall survival.
- Overall and disease-free survival differed across pathological cancer stages.

## Introduction

Esophageal cancer is associated with high morbidity with an overall 5-year survival reported at less than 20% [1–3]. Surgical resection via esophagectomy is an important component of the multimodality treatment approach to esophageal cancer [4, 5]. Minimally invasive esophagectomy (MIE) has been increasingly utilized over traditional open esophagectomy due to the reported benefits in short-term outcomes, including decreased hospital length of stay, reduced postoperative complications, and enhanced quality of life [4–7]. Additionally, MIE has been suggested to offer improved long-term oncological outcomes of overall survival and disease-free survival [2, 8].

Commonly, different MIE techniques, including Ivor Lewis, McKeown, transhiatal, and hybrid or totally MIE, have often been grouped together to serve as a comparator cohort to open esophagectomies; however, perioperative outcomes differ with individual MIE techniques [3, 9–12]. In particular, minimally invasive Ivor Lewis esophagectomy has been associated with a shorter length of stay, fewer postoperative complications, and lower readmission rates compared to the McKeown approach [3, 10, 11]. As perioperative outcomes vary based on MIE techniques, a distinction in long-term outcomes based on MIE techniques should be explored. Currently, there are a limited number of studies assessing individual MIE techniques and associated long-term oncological outcomes [13].

Our institution qualifies as a safety-net hospital that provides care to an underserved area with a high proportion of Medicare, Medicaid, and/or underinsured patients [14]. In addition, our hospital is the regional referral center for medically vulnerable patients requiring complex cancer surgery. Although previous studies have reported suboptimal outcomes after surgical procedures at safety-net hospitals [15–18], we have reported comparable short-term outcomes to a national sample [19]. However, long-term survival outcomes have not been evaluated after minimally invasive Ivor Lewis esophagectomy at our safety-net institution. Therefore, the aim of this study was to determine the long-term oncological survival rate and factors associated with overall survival in patients with esophageal cancer undergoing a totally minimally invasive Ivor Lewis esophagectomy (MILE) at a safety-net hospital.

## Materials and methods

### Study design

This study was approved by the University of Florida Institutional Review Board as a single-institution retrospective analysis (IRB 201901917). Consecutive patients treated for esophageal cancer by thoracolaparoscopic MILE by the senior author (ZTA) between September 2013 and November 2017 at UF Health in Jacksonville, Florida, were included. During this period, six patients underwent open esophagectomy procedures for proximal esophageal cancer and were excluded. The remaining patients underwent MILE. Data from the medical record was extracted regarding patient demographics, baseline characteristics, procedural details, disease characteristics, postoperative outcomes, and survival outcomes through October 2020.

### Patients’ baseline characteristics

Patient demographics included age, sex, and race. Data regarding comorbid conditions were collected, including body mass index, tobacco use, diabetes mellitus, hyperlipidemia, hypertension, cardiovascular disease, respiratory disease, gastrointestinal disease, renal disease, and other prior malignancies at the time of evaluation for surgery. Baseline performance status was assessed by the Eastern Cooperative Oncology Group (ECOG) and Karnofsky performance status (KPS) score [20, 21]. Preoperative risk assessment factors were prior abdominal surgery and the American Society of Anesthesiologists physical status score. Laboratory data at the time of evaluation for surgery included the presence or absence of anemia, hemoglobin, albumin, platelets, white blood cells, lymphocytes, and neutrophils. Patient complexity was defined by Vizient’s case mix index [22].

The area deprivation index (ADI) was derived at the neighborhood level to measure socioeconomic disadvantage [23]. The state ADI deciles and national ADI percentiles were linked to the most recent patient address in the medical record and the patient address recorded at index hospital encounter, respectively. State ADI scores are based on state-only ADI data, which are ranked from lowest to highest and then divided to form deciles (1–10). National ADI scores encompass ADI scores from across the USA, which are then ranked and divided to form percentiles (1–100). Higher rankings are designated to neighborhoods with more disadvantages, and lower rankings are designated to less disadvantaged neighborhoods. In addition, types of primary and secondary insurance payers at the time of operation were determined and included commercial/private, Medicare traditional/indemnity, Medicare/managed care, Medicaid traditional/indemnity, Medicaid/managed care, and other payer types. Low socioeconomic status (SES) was defined based on primary and secondary insurance payer type and included Medicare payers (primary) and Medicaid payers (secondary) and/or Medicare/Medicaid payers (primary) and charity payers (secondary).

### Procedural details and early adverse events

Procedural details that were measured continuously included operative time, estimated blood loss, intraoperative fluid replacement, and urine output. Binary procedural information included conversion to open thoracotomy. Our operative technique for MILE has previously been reported [24]. Early adverse events included postoperative complications occurring during the index hospital admission and within 30 and 90 days following MILE surgery. Postoperative adverse events occurring during index hospital admission were defined as a composite endpoint of any in-hospital complication categorized by the Clavien-Dindo classification as either minor (I, II, IIIa) or major (IIIb, IV, or V) based on the most severe grade of complication [25, 26]. Thirty- and 90-day events were extracted from the medical record and consisted of reoperations, readmissions, and mortality.

### Disease characteristics

Cancer-specific characteristics included the type of cancer, adenocarcinoma or squamous cell carcinoma (SCC) of the esophagus, location of tumors, and use of neoadjuvant chemoradiotherapy [27]. The neoadjuvant protocol was a 5-week period of concurrent chemoradiotherapy. Paclitaxel and carboplatin were given by intravenous infusion on days 1, 8, 15, 22, and 29. External beam radiation with a total dose of 41.4 Gy was given in 23 fractions of 1.8 Gy, 5 fractions a week. Disease severity was defined by the postoperative pathological tumor-node-metastasis (pTNM) staging classification by the American Joint Committee on Cancer/Union for International Cancer Control 8th edition [28, 29]. Other histopathological disease characteristics included pathological complete response indicating no detected viable tumor cells in the resected specimen, the number of lymph nodes harvested, a radical (R0) or positive resection margin, and the presence or absence of lymphovascular invasion. Postoperative development of disease recurrence was categorized as locoregional and/or distant metastasis of disease. Locoregional sites included anastomotic site and locoregional lymph nodes. Distant metastatic sites were the brain, bone, peritoneum, colon, adrenal gland, liver, and lung.

### Follow-up

Regular clinical evaluation occurred in patients after discharge in conjunction with medical oncology. Within the first postoperative year, evaluations occurred every 3 months. Subsequently, follow-up occurred every 6 months in the second and third year and then annually in the fourth and fifth year.

### Survival outcomes

The primary outcomes of interest in this study were patient overall survival (OS) and disease-free survival (DFS). DFS was defined as the time from surgical intervention to the time of disease recurrence or death.

### Statistical analysis

Descriptive statistics were used to characterize the study sample. Categorical variables were summarized as frequencies and counts and analyzed by Pearson’s chi-square (*χ*^2^) tests or Fisher’s exact tests. Continuous variables were summarized as mean and standard deviation or median and interquartile range (IQR), as appropriate. Parametric and nonparametric continuous measures were analyzed by the two-sample *t*-test and Mann-Whitney *U*-test, respectively. *P*-values of < 0.05 defined statistical significance (two-sided).

OS and DFS were reported using Kaplan-Meier estimates. The log-rank test was used to evaluate significant differences in OS and DFS by stage (0–IV) and cancer type (adenocarcinoma or SCC). Patient demographic factors, baseline comorbidities, cancer characteristics, procedural details, and early events were included in a Cox proportional hazard regression model to examine for associations with overall survival. Factors significantly associated with overall survival were incorporated into a multivariable Cox proportional hazard regression model. Hazard ratio (HR), 95% confidence intervals (CI), and *p*-values were reported, and *p* < 0.05 defined statistical significance.

We observed a sharp decrease in the probability of survival until 2 years post-surgery, after which the curve flattened, and there was not a significant difference in mortality between the first and second year. Therefore, we conducted an additional Cox proportional hazard regression model including time-dependent covariates to specifically examine factors associated with mortality within the first 2 years after MILE. Statistical analyses were performed using SAS® University Edition 9.4 (SAS Institute, Cary NC, USA) and IBM® SPSS® statistics 26.0 (IBM, Armonk, NY, USA).

## Results

### Baseline patient characteristics

There were 96 patients treated for esophageal malignancy by MILE between September 2013 and November 2017 (Table 1). The median age was 68.0 years (IQR 60.3–72.0 years) with 77 male patients (80%). The median KPS score was 90.0 (IQR 81.3–90.0), and 62 patients (65%) had an ECOG score ≥ 1. Twelve patients (12%) were categorized within a state ADI decile ≥ 9, whereas seven patients (7%) were categorized within a national ADI percentile ≥ 90 based on index hospital encounter address. The median state ADI (*p* = 0.92) and national ADI (*p* = 0.72) did not significantly differ when comparing the most recent patient address and index encounter address. Twenty-five patients (26%) were covered by private/commercial insurance primary payers, whereas 65 (68%) and six (6%) patients were covered by government-sponsored or other insurance types, respectively. Ten patients (10%) were characterized as low SES based on insurance payer type. Of these, three patients were characterized within a state ADI decile 1–5, three patients as state ADI decile 7, and four patients within the most disadvantaged state ADI decile ≥ 9 based on index encounter address.

**Table 1:** Baseline patient characteristics of 96 patients treated with minimally invasive Ivor Lewis esophagectomy

	All patients ***N*** = 96
	*n* (percent, %) or median [interquartile range] or mean ± standard deviation
***Demographics***	
Age	68.0 [60.3–72.0]
Male	77 (80)
Race	
White	89 (93)
Black/African American	7 (7)
***Comorbidities***	
Body mass index, BMI	25.5 [23.3–28.4]
Any tobacco use	77 (80)
Diabetes mellitus	16 (17)
Hyperlipidemia	32 (33)
Hypertension	52 (54)
Cardiovascular disease	23 (24)
Respiratory disease	36 (38)
Gastrointestinal disease	50 (52)
Renal disease	4 (4)
Other prior malignancies	9 (9)
***Performance status***	
Karnofsky performance status (out of 100)	90.0 [81.3–90.0]
ECOG ≥ 1 performance status	62 (65)
***Preoperative risk assessment***	
Prior abdominal surgery	67 (70)
ASA 1	0
ASA 2	9 (9)
ASA 3	85 (89)
ASA 4	2 (2)
***Laboratory values***	
Anemia	61 (64)
Hemoglobin (g/dL)	12.3 [11.4–13.2]
Albumin (g/dL)	4.0 [3.7–4.2]
Platelets (× 10^9^/L)	206.5 [168.0–244.8]
White blood cells (× 10^9^/L)	5.8 ± 1.9
Lymphocytes (× 10^9^/L)	1.0 [0.7–1.8]
Neutrophils (× 10^9^/L)	4.9 [3.3–6.5]
***Case mix index, CMI***	
Median CMI	2.6 [2.1–2.7]
***Area deprivation index, ADI***	
State ADI decile (1–10) based on most recent address	4.0 [2.0–7.0]
State ADI decile ≥ 9	11 (12)
State ADI decile based on index hospital encounter address	4.0 [2.0–7.0]
State ADI decile ≥ 9	12 (13)
National ADI percentile (1–100) based on most recent address	50.0 [32.0–71.0]
National ADI percentile ≥ 90	7 (7)
National ADI percentile based on index hospital encounter address	49.5 [28.0–71.0]
National ADI percentile ≥ 90	7 (7)
***Insurance primary payer type***	
Private/commercial	25 (26)
Medicare/managed care	21 (22)
Medicare traditional/indemnity	38 (40)
Medicaid/managed care	3 (3)
Medicaid traditional/indemnity	3 (3)
Other primary payer types	6 (6)
Low socioeconomic status based on payer type	10 (10)

*ASA* American Society of Anaesthesiologists physical status score, *ECOG* Eastern Cooperative Oncology Group

### Procedural details

The median operative time was 360.0 min (IQR 325.0–420.0 min; Table 2). Conversion to open thoracotomy was required in 4 patients (4%) due to loss of thoracic domain in obese patients (*n* = 2) and dense mediastinal adhesions (*n* = 1). The other patient required conversion to an emergent thoracotomy for intraoperative bleeding due to an aberrant branch of the pulmonary vein, in which the bleeding was controlled, and the anastomosis was delayed until 48 h later. There was no intraoperative mortality.

**Table 2:** Procedural details and postoperative adverse events of 96 patients treated with minimally invasive Ivor Lewis esophagectomy

	All patients ***N*** = 96
	*n* (percent, %) or median [interquartile range] or mean ± standard deviation
***Procedural details***	
Operative time (minutes)	360.0 [325.0–420.0]
Estimated blood loss (liters)	0.10 [0.05–0.20]
Intraoperative fluid replacement (liters)	2.75 [2.25–3.50]
Urine output (liters)	0.41 [0.30–0.60]
Conversion to open thoracotomy	4 (4)
***Total hospital length of stay***	
Index admission length of stay (days)	8.0 [7.0–10.0]
***Postoperative adverse events during index admission***	
Reoperation	5 (5)
Clavien-Dindo major complications	18 (19)
Anastomotic leak	5 (5)
Graft necrosis	1 (1)
Conduit volvulus/redundancy/obstruction	4 (4)
Respiratory failure requiring intubation	5 (5)
Atrial fibrillation requiring cardioversion	3 (3)
In-hospital mortality	1 (1)
Clavien-Dindo minor complications	27 (28)
Atrial fibrillation	14 (15)
Ventricular tachycardia	1 (1)
Respiratory insufficiency	1 (1)
Pneumonia	1 (1)
Pleural effusion	2 (2)
Pneumothorax	1 (1)
Ileus	1 (1)
Colonic pseudo-obstruction	1 (1)
Clostridium difficile infection	2 (2)
Bacteremia	1 (1)
Urinary retention	1 (1)
Acute kidney injury	1 (1)
***30-day adverse events***	
30-day mortality	2 (2)
30-day readmissions	7 (7)
30-day reoperations	7 (7)
***90-day adverse events***	
90-day mortality	3 (3)
90-day readmissions	8 (8)
90-day reoperations	8 (8)

### Early adverse events

Clavien-Dindo major and minor complications occurred in 18 patients (19%) and 27 patients (28%), respectively (Table 2). A postoperative anastomotic leak occurred in five patients (5%), which were all managed by endoscopic stent placement. Mortality occurred in two patients (2%) within 30 days and an additional one patient (1%) within 90 days.

### Disease characteristics

Adenocarcinoma was present in 75 patients (78%) and SCC in 21 patients (22%) (Table 3). Esophageal tumors were most commonly present in the distal esophageal (*n* = 70), followed by the mid-distal esophagus (*n* = 19) and infrequently seen in the mid-esophagus (*n* = 7). There were 23 patients (24%), 24 patients (25%), 23 patients (24%), 20 patients (21%), and 6 patients (6%) classified as stage 0, I, II, III, and IVA, respectively. Stage IVA was defined in six patients, five of which had N3 disease (seven or more involved lymph nodes), while the sixth patient had an intraoperative finding of involvement of the thoracic aorta (T4b) without nodal involvement. Eighty-eight patients (92%) underwent neoadjuvant chemoradiotherapy. Neoadjuvant treatment was not used in 6 patients with early esophageal cancer, while 2 patients refused treatment. Pathological complete response was achieved in 11 patients (12%). Ninety-four patients (98%) had an R0 resection margin.

**Table 3:** Disease characteristics of 96 patients treated with minimally invasive Ivor Lewis esophagectomy

	All patients ***N = 96***
	*n* (percent, %) or median [interquartile range] or mean ± standard deviation
Type of cancer	
Adenocarcinoma	75 (78)
Squamous cell carcinoma	21 (22)
Neoadjuvant chemoradiotherapy	88 (92)
***Postoperative histopathology***	
Pathological tumor-node-metastasis staging	
Stage 0	23 (24)
Stage I	24 (25)
Stage II	23 (24)
Stage III	20 (21)
Stage IVA	6 (6)
Number of lymph nodes harvested	19.0 [15.0–23.0]
Pathological complete response	11 (12)
R0 resection margin	94 (98)
Positive resection margin	2 (2)
Lymphovascular invasion	
Present	25 (26)
Absent	48 (50)
Unknown	23 (24)

### Survival

The median follow-up time was 33.0 months (IQR 16.3–49.0 months). Only one patient was lost to follow-up. Cancer recurrence was present in 23 patients (24%), of which four patients (all with locoregional recurrence) were still alive at follow-up. Thirteen patients (14%) had locoregional recurrence, 17 patients (18%) had distant metastasis, and 7 patients (7%) had both locoregional and distant recurrence.

**Fig. 1:**
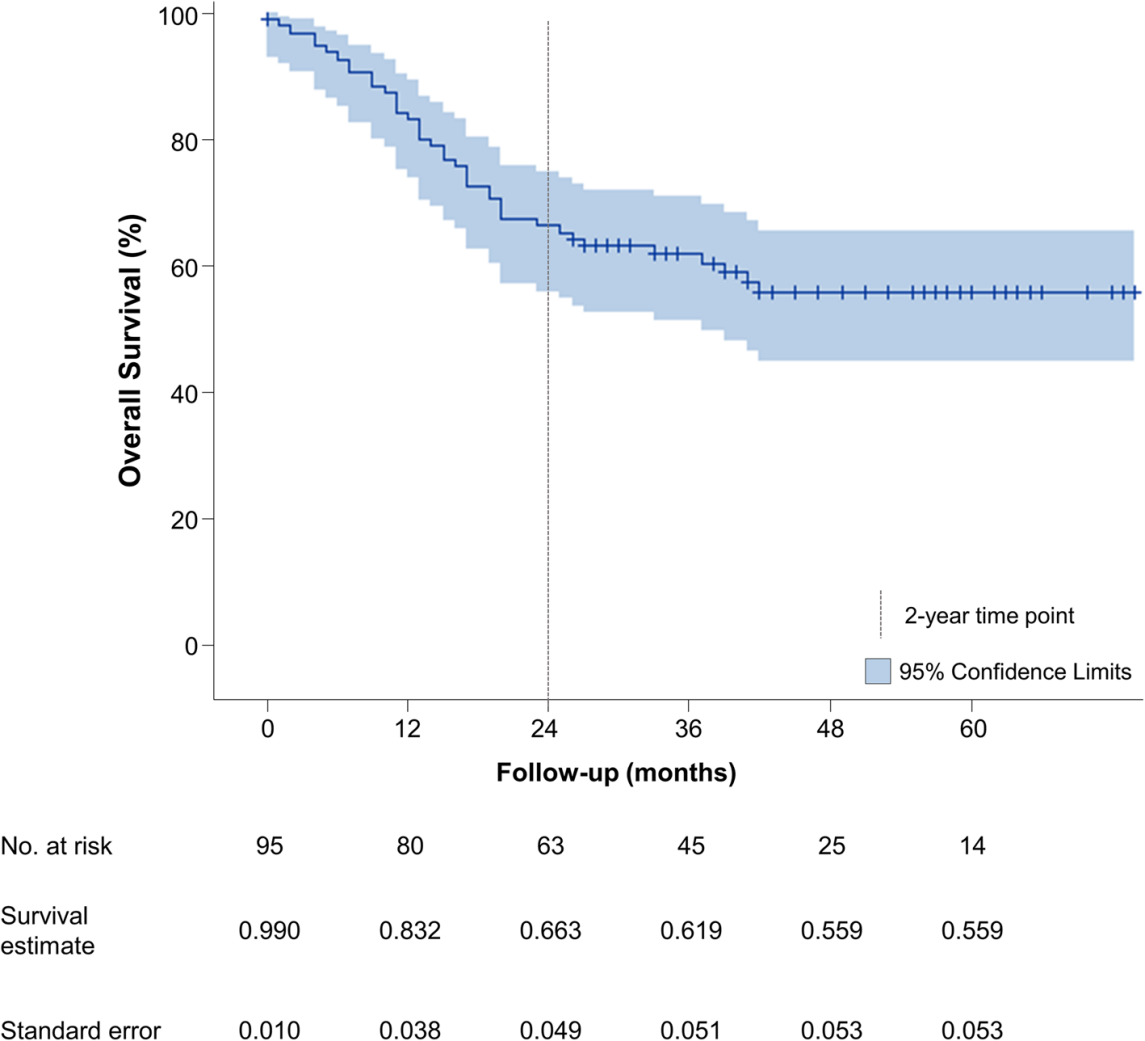
Kaplan-Meier estimates of overall survival in 96 patients

Patient OS at 1, 2, 3, and 5 years following MILE was 83.2% (95% CI, 74.0–89.3%), 66.3% (95% CI, 55.9–74.9%), 61.9% (95% CI, 51.3–70.8%), and 55.9% (95% CI, 44.9–65.6%), respectively (Fig. 1). After stratifying by stage, 5-year OS was 77.3%, 76.7%, 44.7%, 40.5%, and 0% for stage 0, I, II, III, and IV, respectively. One-, 2-, 3-, and 5-year OS stratified by stage are summarized in Table 4 and Fig. 3A. For adenocarcinoma, OS at 3 and 5 years was 61.0% (95% CI, 48.9–71.1%) and 53.8% (95% CI, 41.3–64.8%), respectively. There was no statistical significance (*p* = 0.58) in the OS for adenocarcinoma and SCC (Fig. 4A).

**Table 4:** aplan-Meier estimates of overall survival and disease-free survival stratified by pathological stage 0 to IV

	Stage 0	Stage I	Stage II	Stage III	Stage IV
	Survival percent, % (95% confidence interval)				
***Overall survival***					
1 year	90.9 (68.3–97.6)	87.0 (64.8–95.6)	73.9 (50.9–87.3)	85.0 (60.4–94.9)	83.3(27.3–97.5)
2 years	77.3 (53.7–89.8)	82.6 (60.1–93.1)	52.2 (30.5–70.0)	70.0 (45.1–85.3)	16.7 (0.8–51.7)
3 years	77.3 (53.7–89.8)	82.6 (60.1–93.1)	52.2 (30.5–70.0)	54.0 (30.0–73.0)	0
5 years	77.3 (53.7–89.8)	76.7 (52.3–89.7)	44.7 (22.6–64.7)	40.5 (18.1–62.0)	0
***Disease-free survival***					
1 year	90.9 (68.3–97.6)	87.0 (64.8–95.6)	73.9 (50.9–87.3)	85.0 (60.4–94.9)	83.3 (27.3–97.5)
2 years	77.3 (53.7–89.8)	82.6 (60.1–93.1)	52.2 (30.5–70.0)	70.0 (45.1–85.3)	16.7 (0.8–51.7)
3 year	71.8 (47.4–86.3)	82.6 (60.1–93.1)	52.2 (30.5–70.0)	54.0 (30.0–73.0)	0
5 year	71.8 (47.4–86.3)	76.7 (52.3–89.7)	44.7 (22.6–64.7)	16.2 (2.9–39.4)	0

**Fig. 2:**
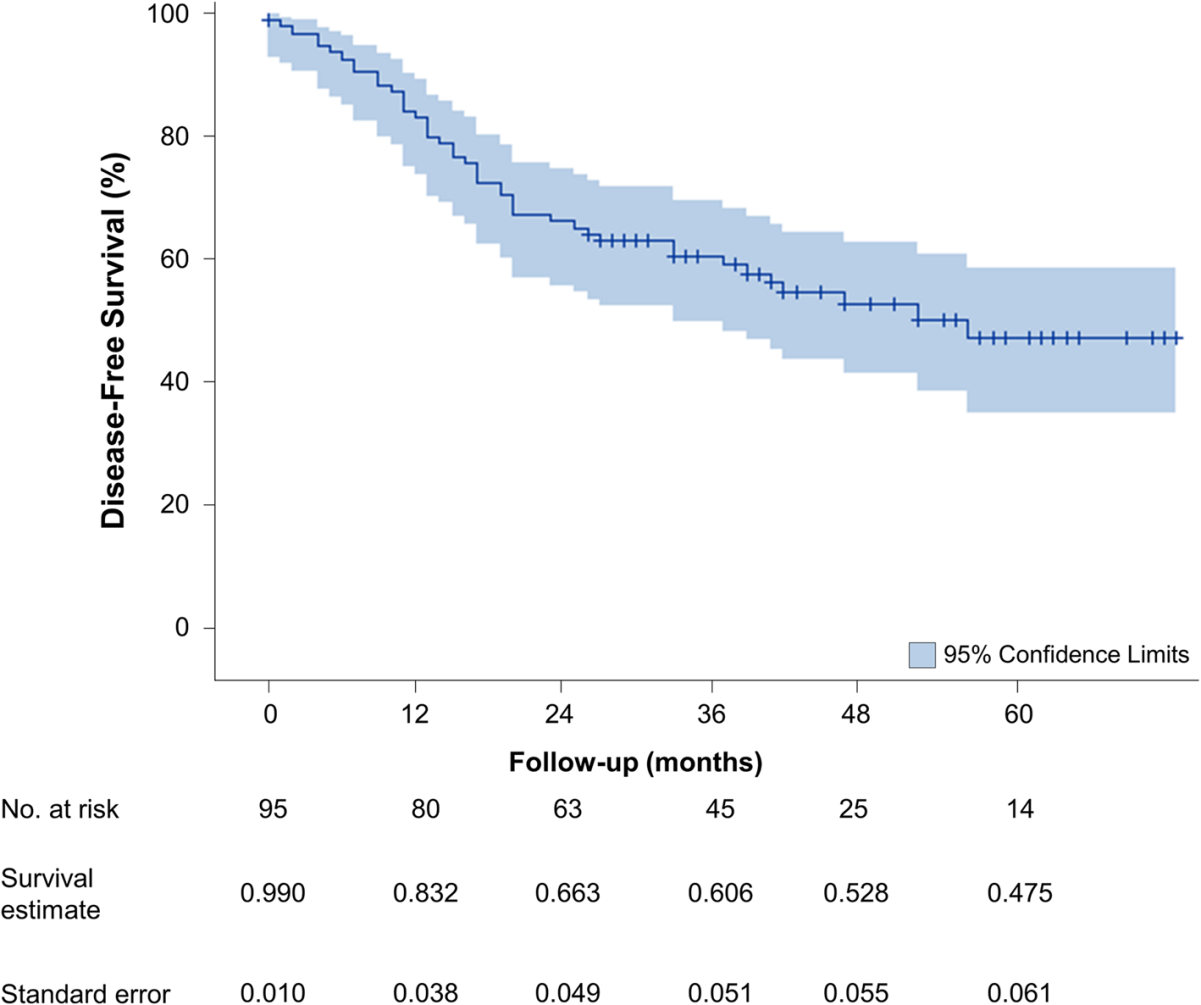
Kaplan-Meier estimates of disease-free survival in 96 patients

**Fig. 3:**
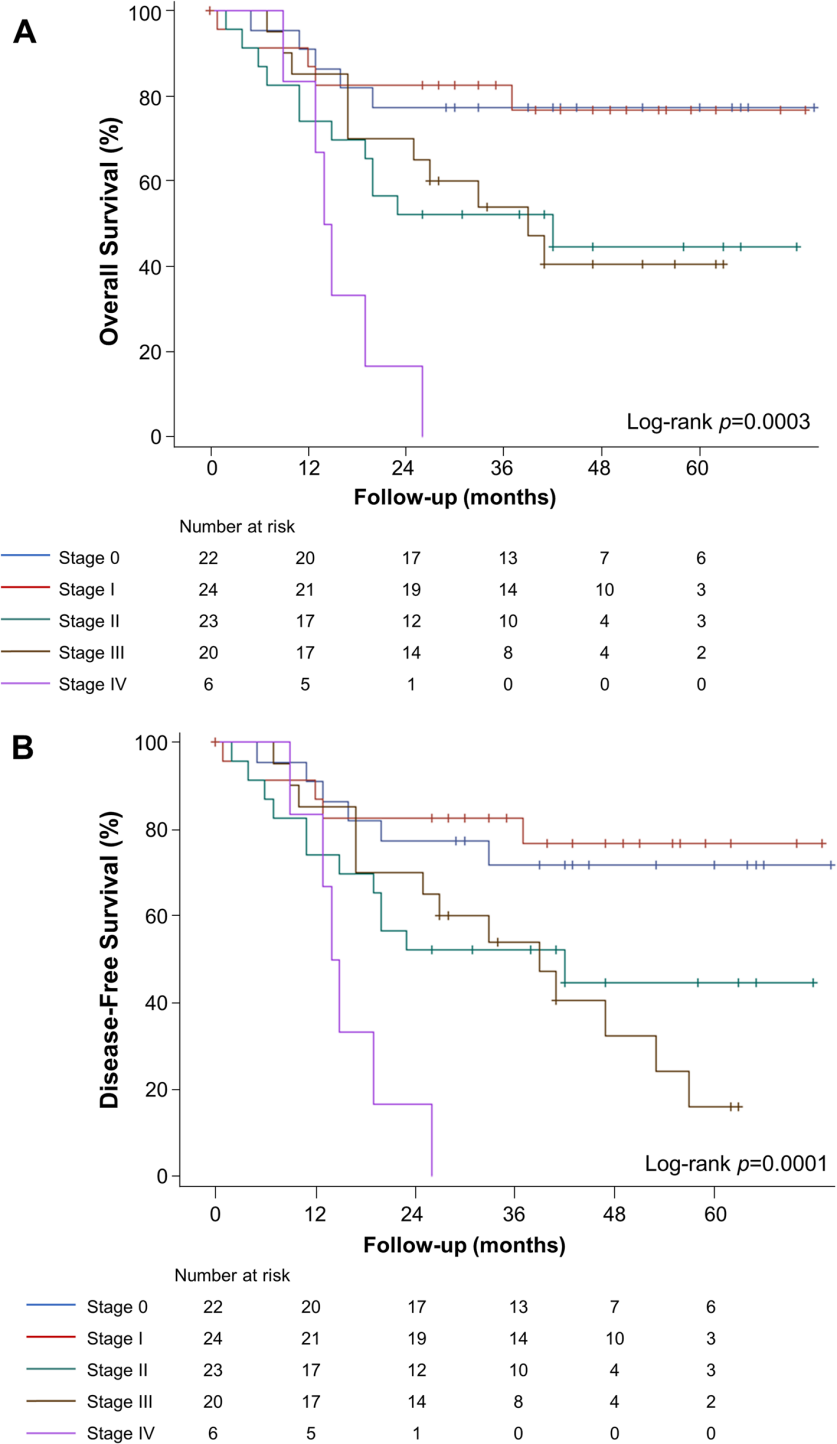
Kaplan-Meier estimates of (**A**) overall survival and (**B**) disease-free survival stratified by pathological stage 0–IV

**Fig. 4:**
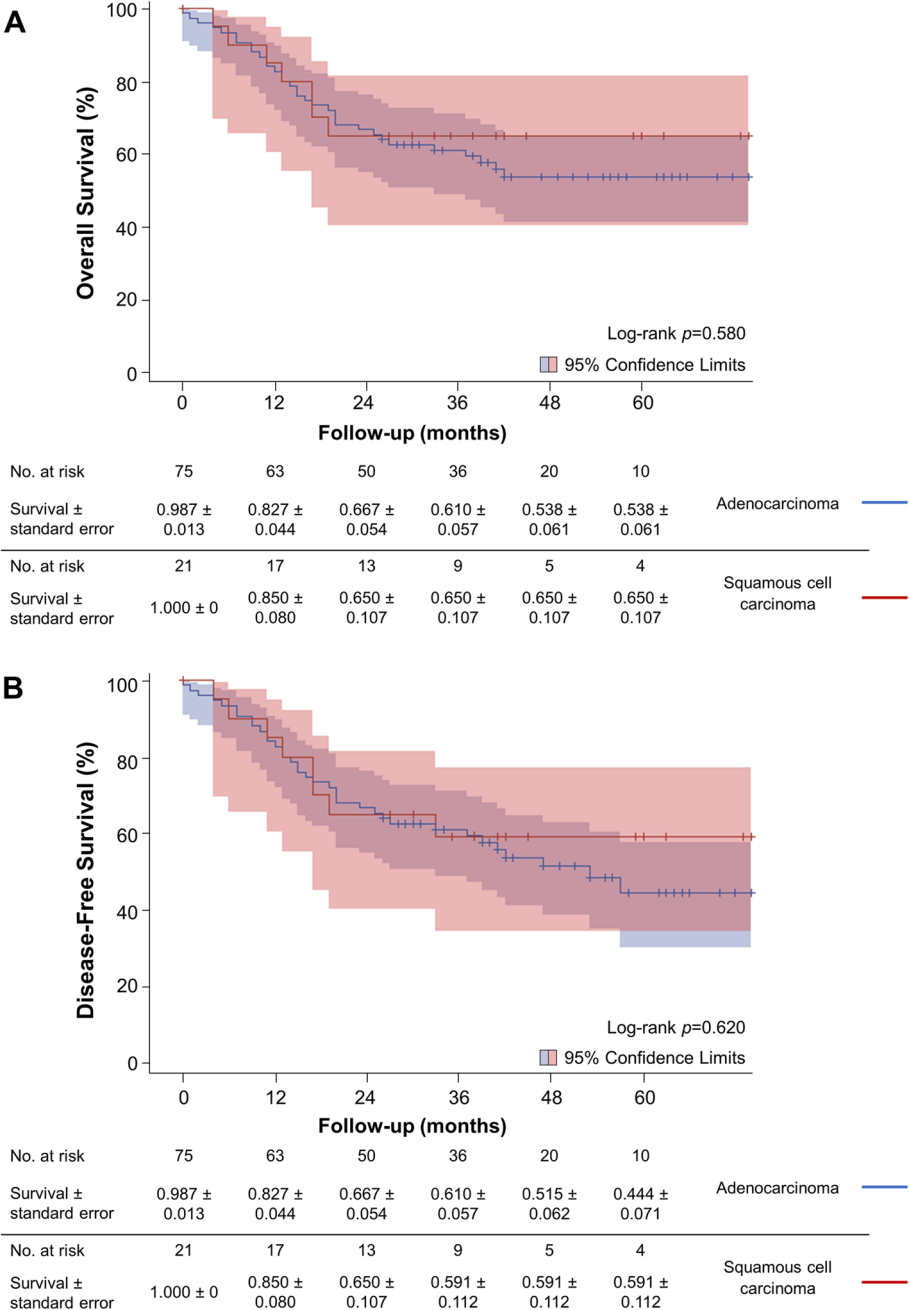
Kaplan-Meier estimates of (**A**) overall survival and (**B**) disease-free survival stratified by esophageal cancer type

DFS at 1, 2, 3, and 5 years following MILE was 83.2% (95% CI, 74.0–89.3%), 66.3% (95% CI, 55.9–74.9%), 60.6 (95% CI, 49.9–69.7%), and 47.5% (95% CI, 35.1–58.8%), respectively (Fig. 2). After stratifying by stage, 5-year DFS was 71.8%, 76.7%, 44.7%, 16.2%, and 0% for stage 0, I, II, III, and IV, respectively. One-, 2-, 3-, and 5-year DFS stratified by stage are summarized in Table 4 and Fig. 3B. OS (*p <* 0.001), and DFS (*p* < 0.001) differed significantly across stages 0 to IV. For adenocarcinoma, 3- and 5-year DFS was 61.0% (95% CI, 48.9–71.1%) and 44.4% (95% CI, 30.2–57.6%; Fig. 4B), respectively. There was no significant difference in DFS among adenocarcinoma and SCC (*p* = 0.62).

On multivariable Cox regression analysis, independent factors negatively affecting OS included increasing age (HR, 1.06; 95% CI, 1.01–1.11; *p* = 0.02), decreasing KPS score (HR, 0.94; 95% CI, 0.90–0.98; *p* = 0.002), stage IV disease (HR, 5.62; 95% CI, 1.87–16.87; *p* = 0.002), locoregional disease recurrence (HR, 2.94; 95% CI, 1.13–7.67; *p* = 0.03), and distant disease recurrence (HR, 4.78; 95% CI, 2.10–10.89; *p* < 0.001; Table 5). All variables included in the Cox regression analysis were displayed in Supplementary Table 1.

**Table 5:** Factors affecting overall survival by univariable and multivariable Cox proportional hazard model regression analysis in 96 patients

	Univariable		Multivariable	
	Unadjusted HR (95% CI)	***p***-value	Adjusted HR (95% CI)	***p***-value
Age	1.049 (1.006–1.093)	0.023	1.060 (1.009–1.114)	0.022*
ASA 4	7.801 (1.751–34.748)	0.007	0.676 (0.099–4.623)	0.690
White blood cell count	1.241 (1.066–1.445)	0.005	1.203 (0.981–1.427)	0.078
Karnofsky performance status	0.959 (0.929–0.991)	0.011	0.937 (0.900–0.976)	0.002*
Low socioeconomic status	2.590 (1.130–5.933)	0.024	1.410 (0.481–4.134)	0.531
pTNM stage IV	4.633 (1.883–11.400)	0.001	5.615 (1.869–16.872)	0.002*
Presence of lymphovascular invasion	2.040 (1.038–4.010)	0.04	0.727 (0.270–1.961)	0.529
Clavien-Dindo minor complications	2.308 (1.229–4.332)	0.009	0.695 (0.278–1.737)	0.437
Locoregional recurrence	2.256 (1.071–4.752)	0.032	2.943 (1.129–7.670)	0.027*
Distant recurrence	6.534 (3.381–2.626)	< 0.001	4.784 (2.102–10.889)	< 0.001*

*HR*, hazard ratio; *CI*, confidence interval; *pTNM*, pathological tumor-node-metastasis stage; *ASA*, American Society of Anaesthesiologists physical status score. Only significant variables on univariable analysis incorporated into multivariable model and summarized in the table. *Statistically significant values (*p* < 0.05)

Mortality occurred more frequently within the first 2 years following surgery (80%) and decreased after 2 years (20%, *p* < 0.001) with no significant difference in mortality between the first and second year (*p* = 1.00). The Kaplan-Meier curve illustrated a sharp decline in OS until the 2-year timepoint (Fig. 1); therefore, we conducted an additional multivariable Cox proportional hazard regression analysis with time-dependent covariate predicting factors associated with 2-year survival. An independent association with 2-year mortality was seen with stage IV disease (HR, 3.29; 95% CI, 1.04–10.41; *p* = 0.04) and distant disease recurrence (HR, 5.78; 95% CI, 2.61–12.81; *p* < 0.001; Table 6).

**Table 6:** Significant factors affecting overall survival within 2 years by univariable and multivariable Cox proportional hazard model regression analysis

	Univariable		Multivariable	
	Unadjusted HR (95% CI)	***p***-value	Adjusted HR (95% CI)	***p***-value
Karnofsky performance status	0.955 (0.924–0.988)	0.007	0.959 (0.916–1.003)	0.070
ECOG performance status ≥ 1	2.715 (1.117–6.601)	0.028	2.849 (0.908–8.936)	0.073
National ADI≥ 90 based on index encounter address	3.266 (1.136–9.388)	0.028	0.374 (0.049–2.834)	0.341
Low socioeconomic status	2.477 (1.009–6.079)	0.048	1.131 (0.397–3.225)	0.818
pTNM stage IV	3.801 (1.441–10.026)	0.007	3.292 (1.042–10.408)	0.042*
Clavien-Dindo minor complications	2.254 (1.120–4.538)	0.023	2.063 (0.958–4.442)	0.064
Locoregional recurrence	2.029 (0.876–4.699)	0.099	1.244 (0.442–3.501)	0.679
Distant recurrence	5.430 (2.659–11.089)	< 0.001*	5.784 (2.612–12.810)	<0.001*

*HR*, hazard ratio; *CI*, confidence interval; *ECOG*, Eastern Cooperative Oncology Group; *pTNM*, pathological tumor-node-metastasis stage. Only significant variables on univariable analysis incorporated into multivariable model and summarized in the table. *Statistically significant values (*p* < 0.05)

National ADI ≥ 90 based on index hospital encounter was not predictive of OS (HR, 2.52; 95% CI, 0.89–7.12; *p* = 0.08) and was not associated with 2-year survival (HR, 0.36; 95% CI, 0.0–2.74; *p* = 0.33). Additionally, low SES based on insurance payer types was not associated with OS (HR, 1.41; 95% CI, 0.48–4.13; *p* = 0.53) nor 2-year survival (HR, 1.13; 95% CI, 0.40–3.23; *p* = 0.82).

## Discussion

MIE has been increasingly adopted as short-term outcomes have been comparable to open esophagectomies [4–6, 30–32]. Long-term outcomes after individual MIE techniques, particularly for MILE, have not been well-described in the literature. This study is novel as it provides insight into these long-term cancer survival outcomes in a cohort of MILE-only patients at a safety-net hospital.

In our study, OS at 3 and 5 years was 62% and 56%, and DFS at 3 and 5 years was 61% and 48%, respectively. In a recent study, Veenstra and colleagues [33] reported a 3-year DFS of 63% after McKeown MIE for adenocarcinoma or SCC in a sample of 106 patients. In a follow-up study of the TIME trial [4, 30], 3-year DFS and OS was 43% and 43%, respectively, in a sample of 59 patients undergoing either McKeown MIE or MILE for adenocarcinoma or SCC. Our outcomes correspond to the reported rates of minimally invasive approaches, therefore suggesting favorable oncological survival after MILE at our institution. Notably, a study comparing OS and DFS between McKeown and thoracolaparoscopic esophagectomy techniques found no significant difference between survival rates [13]. However, the sample size of each cohort was small with *n* = 51 in the thoracolaparoscopic group and *n* = 62 in the McKeown group, and 46% of patients died during the follow-up period. Additionally, no patients received neoadjuvant chemotherapy in this study. In contrast, a significant proportion of our patients (92%) underwent neoadjuvant concurrent chemoradiation, which may be an additional factor contributing to our favorable survival rates. In a recent meta-analysis, Kumar and colleagues report a significant survival benefit at 3-year OS (*p* < 0.01*)*, 3-year DFS (*p* < 0.001), and 5-year DFS (*p* < 0.001) in those patients who underwent neoadjuvant concurrent chemoradiation over upfront surgery [34].

We observed a significant difference in survival across stages as more advanced stages (II, III, IV) had decreased survival compared to stages 0 and I at 5 years. Notably, we observed survival contradiction between stages II and III at the 2-year period. This could not be explained by stage migration nor when the weight of the N stage may be overestimated, while the T stage is underestimated simultaneously as at the 5-year period, stage II OS exceeded stage III OS. Similarly, OS for stage I was greater than that of stage 0 at 2 years; however, stage 0 had better OS than stage I at 5 years.

Few studies have assessed long-term survival based on pTNM stage in MIEs [35–37]. Akin to our series, OS has been shown to decrease with advancing TNM stage when several minimally invasive approaches have been used, including Ivor Lewis, transhiatal, and McKeown, as well as hybrid or totally minimally invasive techniques [36–38]. We further determined independent factors associated with poor OS to be increasing age, decreasing KPS scores, stage IV disease, and the development of locoregional and distant recurrence. Age has been previously reported as a predictor of overall survival in thoracoscopic esophagectomies [39–41]. Patients’ baseline comorbidities are known to impact postoperative outcomes, and we further report baseline performance status as assessed by KPS score to independently affect long-term overall survival outcomes. Not unexpectedly, stage IV disease and the development of disease recurrence in our series at any timepoint after surgical resection were associated with an abridged survival rate. Interestingly, Wang and colleagues reported unresected small lymph nodes observed on postoperative CT, along with tumor grade, and poor differentiation was associated with poor prognostic factors of OS for pT3N0M0 thoracic esophageal squamous cell carcinoma [42].

Moreover, we noted a significant decline in OS within the first 2 years, after which survival somewhat plateaued. As such, we examined factors independently associated with 2-year mortality and found those to be the presence of stage IV disease and development of distant recurrence. Based on our multivariable Cox regression analysis, we could not identify additional factors directly associated with the decline in 2-year survival. Factors affecting 2-year survival were congruent with factors affecting long-term survival (5 years). Notably, postoperative complications did not independently affect 2-year survival nor overall survival in our series. Further research efforts may be needed to explore the effects of esophageal cancer after MILE on early survival outcomes.

ADI scores and a low SES based on insurance payer types were not independently associated with survival in our series. This lack of association may arise from the ADI being derived from a general assessment of a neighborhood and, therefore, does not account for patient-level factors affecting deprivation. Given the temporal nature of the study outcome, changes over time in individual socioeconomic factors may not be captured by a cross-sectional neighborhood ADI. Furthermore, the majority of patients were classified with a national ADI of less than 90 suggesting less neighborhood disadvantage. Interestingly, there were patients characterized as low SES based on insurance payer types, however, fell into a higher socioeconomic group based on ADI. This disparity may reflect the diversity in individual and environmental factors that influence SES, which may not be exclusively described by proxy measures of neighborhood-level deprivation or insurance payer types alone. Nonetheless, MILE at our safety-net hospital provides favorable long-term outcomes to medically high-risk patients, and outcomes are not to be influenced by patients’ neighborhood SES nor medical insurance type.

### Limitations

This study presents several limitations. Given the retrospective design, there are limitations in the availability of data used to assess effects on survival, therefore introducing a confounding effect on the current factors associated with survival. However, we attempt to correct this by using other variables in our models including TNM stage, case mix index, ADI, and insurance payer types. ADI and insurance payer types may not entirely reflect individual socioeconomic status, however, provides a useful measure to help identify generalized disadvantaged groups, particularly at a safety-net hospital. Additionally, statistical outcomes should be carefully deliberated due to the relatively small sample size, although our sample size is comparable to other studies among the population of MIEs. Generalization of outcomes from our series may be restricted due to a single-institution analysis from an individual surgeon; therefore, further studies with larger sample sizes may be valuable in investigating intricacies, including potential confounding factors, which could not be observed in our study. The esophagectomy program at our institution began in 2013 by the senior author (ZTA). Since its inception, all esophagectomies have been performed using minimally invasive techniques; therefore, open or other comparator groups were not available at our institution. Nonetheless, we believe our study adds value in evaluating long-term outcomes after totally MILE in a high-risk patient population at a safety-net hospital and explores the need for future studies to examine survival outcomes after individual MIE approaches.

## Conclusion

Treatment of esophageal cancer by MILE is a safe procedure with a low rate of perioperative complications at our safety-net hospital. Furthermore, MILE offers favorable long-term oncological OS and DFS, particularly for patients with less advanced cancer stages. Increasing age, decreasing KPS scores, the presence of pTNM stage IV disease, and the development of locoregional and distant disease recurrence are shown to be prognostic factors impacting overall survival. Prospective studies comparing long-term outcomes after different MIE approaches are warranted to validate survival outcomes after MILE.

## Supplementary Information

**Additional file 1:** **Table S1. **All variables used to assess overall survival by univariable and multivariable Cox proportional hazard model regression analysis in 96 patients.

## Data Availability

The datasets used and/or analyzed during the current study are available from the corresponding author on reasonable request.

## Abbreviations

MIE: Minimally invasive esophagectomy
MILE: Minimally invasive Ivor Lewis esophagectomy
ECOG: Eastern Cooperative Oncology Group
KPS: Karnofsky performance status
ADI: Area deprivation index
SES: Socioeconomic status
SCC: Squamous cell carcinoma
pTNM: Pathological tumor-node-metastasis
OS: Overall survival
DFS: Disease-free survival
IQR: Interquartile range
HR: Hazard ratio
CI: Confidence intervals
