# Supplementary material for: Long-term survival outcomes of esophageal cancer after minimally invasive Ivor Lewis esophagectomy
Source: World J Surg Oncol. 2022 Feb 25;20:50. doi: 10.1186/s12957-022-02518-0 (PMC8876443; doi:10.1186/s12957-022-02518-0)
Supplement: Supplementary file 1 — Additional file 1: Table S1. All variables used to assess overall survival by univariable and multivariable Cox proportional hazard model regression analysis in 96 patients. [file 12957_2022_2518_MOESM1_ESM.docx]

**Supplementary Table 1.** All variables used to assess overall survival by univariable and multivariable Cox proportional hazard model regression analysis in 96 patients.

|  | **Univariable** | |  | | **Multivariable** | |  | |  |
| --- | --- | --- | --- | --- | --- | --- | --- | --- | --- |
|  | **Unadjusted HR**  **(95% CI)** | | ***p*-value** | | **Adjusted HR**  **(95% CI)** | | ***p*-value** | |  |
|  |  | |  | |  | |  | |  |
| Age | 1.049 (1.006-1.093) | | 0.023 | | 1.060 (1.009-1.114) | | 0.022* | |  |
| ASA 4 | 7.801 (1.751-34.748) | | 0.007 | | 0.676 (0.099-4.623) | | 0.690 | |  |
| White blood cell count | 1.241 (1.066-1.445) | | 0.005 | | 1.203 (0.981-1.427) | | 0.078 | |  |
| Karnofsky performance status | 0.959 (0.929-0.991) | | 0.011 | | 0.937 (0.900-0.976) | | 0.002* | |  |
| Low socioeconomic status | 2.590 (1.130-5.933) | | 0.024 | | 1.410 (0.481-4.134) | | 0.531 | |  |
| pTNM stage IV | 4.633 (1.883-11.400) | | 0.001 | | 5.615 (1.869-16.872) | | 0.002* | |  |
| Presence of lymphovascular invasion | 2.040 (1.038-4.010) | | 0.04 | | 0.727 (0.270-1.961) | | 0.529 | |  |
| Clavien-Dindo minor complications | 2.308 (1.229-4.332) | | 0.009 | | 0.695 (0.278-1.737) | | 0.437 | |  |
| Locoregional recurrence | 2.256 (1.071-4.752) | | 0.032 | | 2.943 (1.129-7.670) | | 0.027* | |  |
| Distant recurrence | 6.534 (3.381-12.626) | | <0.001 | | 4.784 (2.102-10.889) | | <0.001* | |  |
| Male | 1.497 (0.628-3.568) | | 0.362 | |  | |  | |  |
| White race | 0.6690 (0.238-1.883) | | 0.446 | |  | |  | |  |
| Body mass index | 1.497 (0.628-3.568) | | 0.362 | |  | |  | |  |
| Any tobacco use | 1.117 (0.494-2.528) | | 0.790 | |  | |  | |  |
| Diabetes mellitus | 1.642 (0.782-3.450) | | 0.190 | |  | |  | |  |
| Hyperlipidemia | 1.301 (0.686-2.469) | | 0.421 | |  | |  | |  |
| Hypertension | 1.519 (0.801-2.883) | | 0.201 | |  | |  | |  |
| Any cardiovascular disease | 1.798 (0.927-3.486) | | 0.082 | |  | |  | |  |
| Any respiratory disease | 0.949 (0.495-1.820) | | 0.876 | |  | |  | |  |
| Any gastrointestinal disease | 0.516 (0.274-0.973) | | 0.061 | |  | |  | |  |
| Any renal disease | 0.561 (0.077-4.091) | | 0.569 | |  | |  | |  |
| Other prior malignancies | 1.451 (0.567-3.711) | | 0.437 | |  | |  | |  |
| ECOG performance status ≥ 1 | 2.257 (0.974-4.746) | | 0.062 | |  | |  | |  |
| Prior abdominal surgery | 0.910 (0.469-1.764) | | 0.780 | |  | |  | |  |
| ASA 2 | 0.695 (0.214-2.253) | | 0.544 | |  | |  | |  |
| ASA 3 | 0.881 (0.345-2.250) | | 0.791 | |  | |  | |  |
| Anemia | 1.231 (0.634-2.391) | | 0.540 | |  | |  | |  |
| Hemoglobin | 0.869 (0.689-1.096) | | 0.236 | |  | |  | |  |
| Albumin | 0.747 (0.370-1.507) | | 0.415 | |  | |  | |  |
| Platelets | 0.998 (0.994-1.003) | | 0.526 | |  | |  | |  |
| Lymphocytes | 1.198 (0.807-1.778) | | 0.371 | |  | |  | |  |
| Neutrophils | 1.171 (0.986-1.391) | | 0.071 | |  | |  | |  |
| Case Mixed Index | 0.973 (0.852-1.112) | | 0.689 | |  | |  | |  |
| State ADI decile ≥ 9 based on most recent address | 1.674 (0.700-4.000) | | 0.247 | |  | |  | |  |
| State ADI decile ≥ 9 based on index hospital encounter address | 1.527 (0.639-3.645) | | 0.341 | |  | |  | |  |
| National ADI percentile (1-100) based on most recent patient address | 1.008 (0.995-1.022) | | 0.221 | |  | |  | |  |
| National ADI percentile ≥ 90 | 2.101 (0.746-5.920) | | 0.160 | |  | |  | |  |
| National ADI ≥ 85 | 1.435 (0.601-3.426) | | 0.417 | |  | |  | |  |
| National ADI percentile (1-100) based on index hospital encounter address | 1.006 (0.993-1.019) | | 0.337 | |  | |  | |  |
| National ADI percentile ≥ 90 | 2.520 (0.892-7.120) | | 0.081 | |  | |  | |  |
| National ADI ≥ 85 | 1.527 (0.639-3.645) | | 0.341 | |  | |  | |  |
| Private/commercial primary insurance payer | 0.883 (0.431-1.807) | | 0.733 | |  | |  | |  |
| Medicare/managed care insurance | 0.860 (0.396-1.868) | | 0.703 | |  | |  | |  |
| Medicare traditional/indemnity insurance | 1.001 (0.531-1.887) | | 0.997 | |  | |  | |  |
| Medicaid/managed care insurance | 2.358 (0.566-9.827) | | 0.239 | |  | |  | |  |
| Medicaid traditional/indemnity insurance | 0.867 (0.119-6.312) | | 0.888 | |  | |  | |  |
| Other primary insurance payers | 0.599 (0.082-4.361) | | 0.613 | |  | |  | |  |
| Operative time | 1.002 (0.999-1.006) | | 0.121 | |  | |  | |  |
| Intraoperative estimated blood loss | 0.758 (0.268-2.144) | | 0.602 | |  | |  | |  |
| Intraoperative fluid replacement | 0.995 (0.759-1.304) | | 0.969 | |  | |  | |  |
| Intraoperative urine output | 0.506 (0.138-1.859) | | 0.305 | |  | |  | |  |
| Conversion to open thoracotomy | 2.205 (0.679-7.164) | | 0.188 | |  | |  | |  |
| Index admission length of stay | 0.977 (0.921-1.036) | | 0.438 | |  | |  | |  |
| Reoperation during index admission | 1.077 (0.259-4.472) | | 0.919 | |  | |  | |  |
| Clavien-Dindo major complications | 0.797 (0.334-1.904) | | 0.610 | |  | |  | |  |
| 30-day readmissions | 2.406 (0.855-6.775) | | 0.096 | |  | |  | |  |
| 30-day reoperations | 1.612 (0.221-11.753) | | 0.638 | |  | |  | |  |
| 90-day readmissions | 1.788 (0.635-5.034) | | 0.271 | |  | |  | |  |
| 90-day reoperations | 0.049 (0-5606.005) | | 0.611 | |  | |  | |  |
| Adenocarcinoma | 1.257 (0.556-2.841) | | 0.583 | |  | |  | |  |
| Squamous cell carcinoma | 0.796 (0.352-1.799) | | 0.583 | |  | |  | |  |
| Neoadjuvant chemoradiotherapy | 1.982 (0.478-8.219) | | 0.346 | |  | |  | |  |
| pTNM stage 0 | 0.419 (0.164-1.071) | | 0.069 | |  | |  | |  |
| pTNM stage I | 0.402 (0.157-1.029) | | 0.057 | |  | |  | |  |
| pTNM stage II | 1.574 (0.797-3.109) | | 0.191 | |  | |  | |  |
| pTNM stage III | 1.454 (0.723-2.923) | | 0.293 | |  | |  | |  |
| Number of lymph nodes harvested | 0.907 (0.852-1.965) | | 0.201 | |  | |  | |  |
| Pathological complete response | 0.164 (0.023-1.197) | | 0.075 | |  | |  | |  |
| Positive resection margin | 3.746 (0.887-15.822) | | 0.072 | |  | |  | |  |
|  | |  | |  | |  | |  | |

Only significant variables on univariable Cox regression analysis incorporated into multivariable model. *HR, Hazard Ratio; CI, Confidence Interval; pTNM, Pathological Tumor-Node-Metastasis stage; ASA, American Society of Anaesthesiologists physical status score; ECOG, Eastern Cooperative Oncology Group; ADI, Area Deprivation Index.*

**Statistically significant values (p<0.05).*
